# Insulin-Like Growth Factor 1 Regulates Acute Inflammatory Lung Injury Mediated by Influenza Virus Infection

**DOI:** 10.3389/fmicb.2019.02541

**Published:** 2019-11-26

**Authors:** Guiping Li, Lijuan Zhou, Can Zhang, Yun Shi, Derong Dong, Miao Bai, Rong Wang, Chuanfu Zhang

**Affiliations:** ^1^Center for Hygienic Assessment and Research, Center for Disease Control and Prevention of Chinese PLA, Beijing, China; ^2^College of Life Science, Huaibei Normal University, Huaibei, China; ^3^Laboratory of Protein Engineering, Beijing Institute of Biotechnology, Beijing, China

**Keywords:** influenza virus, IGF1, acute inflammatory lung injury, PI3K/AKT, MAPK

## Abstract

The acute inflammatory lung injury is an important cause of death due to influenza A virus (IAV) infection. Insulin-like growth factor 1 (IGF1) played an important role in the regulation of inflammation in the immune system. To investigate the role of IGF1 in IAV-mediated acute inflammatory lung injury, the expression of IGF1 and inflammatory cytokines was tested after IAV A/Puerto Rico/8/1934 (H1N1; abbreviated as PR8) infection in A549 cells. Then, a BALB/c mouse model of PR8 infection was established. On days 3, 5, 7, and 9 post-infection, the mice lung tissue was collected to detect the expression changes in IGF1 mRNA and protein. The mice were divided into four groups: (1) PBS (abbreviation of phosphate buffered saline); (2) PR8 + PBS; (3) PR8 + IGF1; and (4) PR8 + PPP (abbreviation of picropodophyllin, the IGF1 receptor inhibitor). The body weight and survival rate of the mice were monitored daily, and the clinical symptoms of the mice were recorded. On day 5 post-infection, the mice were sacrificed to obtain the serum and lung tissues. The expression of inflammatory cytokines in the serum was detected by enzyme linked immunosorbent assay; lung injury was observed by hematoxylin-eosin staining; the viral proliferation in the lung was detected by real-time quantitative PCR; and the protein expression of the main molecules in the phosphatidylinositol-3-kinases/protein kinase B (PI3K/AKT) and mitogen-activated protein kinase (MAPK) signaling pathways was detected by Western blot. It was found that IGF1 expression is upregulated in A549 cells and BALB/c mice infected with PR8, whereas IGF1 regulated the expression of inflammatory cytokines induced by PR8 infection. Overexpression of IGF1 aggravated the IAV-mediated inflammatory response, whereas the inhibition of IGF1 receptor reduced such inflammatory response. The phosphorylation of IGF1 receptor triggered the PI3K/AKT and MAPK signaling pathways to induce an inflammatory response after IAV infection. Therefore, IGF1 plays an important immune function in IAV-mediated acute inflammatory lung injury. IGF1 may provide a therapeutic target for humans in response to an influenza outbreak, and inhibition of IGF1 or IGF1 receptor may represent a novel approach to influenza treatment.

## Introduction

Influenza is an acute infectious disease caused by influenza virus infection, which is primarily characterized by respiratory damage. Moreover, it is associated with serious features (e.g., acute onset, wide spread, strong contagiousness, and great harm), which seriously threatens human health. Studies have shown that a series of symptoms and consequences caused by the influenza virus are not caused by the direct action of the influenza virus itself but are rather due to the inflammatory injury caused by the excessive activation of the immune response induced by an influenza virus infection (Oda et al., 1989; Taubenberger and Morens, 2006; Basler and Aguilar, 2008; Li and Cao, 2017). During the process of an IAV infection, there are different cytokine waves from the initial early responses of monocyte chemotactic protein-1 (MCP-1), interleukin-8 (IL-8), and interferon-alpha/beta/kappa (IFN-α/β/κ) to the later responses of IL-1α/β, IL-6, IL-18, tumor necrosis factor-alpha (TNF-α), IFN type I, macrophage inflammatory protein-1 alpha/beta (MIP-1α/β), MIP-3α, MCP-3, and interferon-inducible protein-10 (IP-10), which are mainly secreted by infected macrophages in the lower respiratory tract (Julkunen et al., 2001; Xagorari and Chlichlia, 2008; Jewell et al., 2010; Gu et al., 2019). The modest cytokine response contributes to the induction of antiviral and Th1-type immune responses in the body; however, excessive inflammatory responses can be harmful. For example, the main reason for the high pathogenicity of the H5N1 avian influenza virus is the exaggerated generation and secretion of excessively high levels of pro-inflammatory cytokines, known as a “cytokine storm” (Kobasa et al., 2007; Allen et al., 2009). In 1918, the “Spanish flu” also caused fatal inflammatory damage to the lung tissue by triggering an overreaction of the human immune response (Ocana-Macchi et al., 2009). This inflammatory injury to the lung tissue is both an important cause of death due to influenza virus infection and a major cause of lung infections caused by severe acute respiratory syndrome (SARS), sepsis, and aspiration pneumonia (Imai et al., 2005; Nicholls and Peiris, 2005). Currently, immunosuppressive agents (e.g., glucocorticoids) are often used in clinical practice to inhibit inflammatory cytokine responses, thereby blocking disease progression and improving the clinical therapeutic effect. The literature reports that 51–69% of patients with severe H1N1 influenza are treated with glucocorticoids, which have a specific therapeutic effect (Aikawa et al., 2012); however, glucocorticoids systemically inhibit the immune function of the infected individual. Moreover, the long-term treatment with such medication can induce or aggravate the infection, causing a variety of complications and serious side effects (e.g., femoral head necrosis). Therefore, it is imperative to develop a deep understanding of the pathogenesis of influenza virus and to identify new strategies of treating influenza more safely and effectively.

Insulin-like growth factor 1 (IGF1) belongs to the insulin-like growth factor family, which also includes growth hormone (GH), insulin-like growth factor II (IGF2), insulin-like growth factor 1 receptor (IGF1R), insulin-like growth factor II receptor (IGF2R), and insulin-like growth factor binding protein 1–6 (IGFBP1–6; Jogie-Brahim et al., 2009). This family plays an extremely important role in the process of cell growth, differentiation, and apoptosis (Stewart and Rotwein, 1996; Granata et al., 2004). IGF1 mainly functions by binding to IGF1R, a transmembrane protein composed of two α domains and two β domains (Siddle et al., 2001). The α domain binds to IGF1 to activate the β domain (Siddle et al., 2001). Since the β domain has tyrosine kinase activity, it can promote the phosphorylation of the substrate hepatocyte growth factor (HGF), docking protein insulin receptor substrate (IRS), vascular endothelial growth factor (VEGF), and growth factor receptor binding protein 2 (Grb2; Wilden et al., 1992; Hubbard, 1997). Some phosphorylated substrates activate the downstream phosphatidylinositol-3-kinases/protein kinase B (PI3K/AKT) and mitogen-activated protein kinase (MAPK) signaling pathways to regulate a range of biological responses (Myers et al., 1992; Egan et al., 1993).

Recent studies have found that IGF1 plays an important role in the regulation of inflammation in the immune system. IGF1 mRNA expression in the bronchial cells of asthmatic patients was significantly higher than that of normal people and was significantly associated with fibrosis in epithelial cells (Hoshino et al., 1998). The associated mechanism is that IGF1 binds to the receptor and activates the PI3K/AKT signaling pathway and induces Akt activation, which further activates the downstream IL-17-mediated inflammatory pathway (Lee et al., 2014). In addition, the levels of serum IGF1 protein in patients with type 2 diabetes are significantly higher than those in healthy people. Obesity is closely related to the pathogenesis of type 2 diabetes, as long-term obesity will lead to IL-6 and IL-17-mediated chronic inflammation (Cohen and LeRoith, 2012; Ge et al., 2013). In non-autoimmune inflammatory contact dermatitis, IGF1 relieves the inflammatory response by recruiting regulatory T cells to release the anti-inflammatory cytokine, IL-10 (Johannesson et al., 2014). In the nervous system, IGF1 recruits anti-inflammatory proteins to protect nerve cells from degeneration (Arroba et al., 2016). Other studies have shown that IGF1 plays an important role in regulating the function of lactating buffalo oocytes and preventing inflammation induced by post-partum genital tract infections. A concentration of 50 ng/ml IGF1 acts on lipopolysaccharides (LPS; 1 μg/ml)-infected buffalo granulosa cells, reducing the expression of the inflammatory cytokines, IL-6, TNF-α, and IL-1β, as well as decreasing Akt phosphorylation in PI3K/AKT signaling pathway and extracellular-regulated kinase 1/2 (ERK1/2) phosphorylation in the MAPK signaling pathway (Onnureddy et al., 2015).

However, whether IGF1 plays a significant role in mediating inflammation and pathology during influenza infection and its associated mechanism remains unknown. In this study, we found that IGF1 mRNA and protein increased after influenza virus infection. Overexpression of IGF1 aggravated cytokine expression during infection by influenza, while blocking of IGF1 production in mice treated with IGF1R inhibitor, decreased immunopathology. The phosphorylation level of IGF1R was elevated after influenza virus infection, triggering the PI3K/AKT and MAPK signaling pathways to induce an inflammatory response. Thus, IGF1 could regulate influenza virus-mediated acute inflammatory lung injury, which may provide a therapeutic target for humans in response to an influenza outbreak.

## Materials and Methods

### Cell Lines, Viruses, and Animals

The human alveolar epithelial cell line A549 is particularly sensitive to influenza virus infection and has been widely used as a good *in vitro* model to study influenza virus for nearly 20 years. The cell line A549 was purchased from the American Type Culture Collection (ATCC, USA) and propagated in Dulbecco’s Modified Eagle’s Medium (DMEM; Life Technologies, USA) supplemented with 10% fetal bovine serum (FBS; HyClone, USA) at 37°C in a 5% CO_2_ incubator.

The mouse adapted Influenza A virus (IAV) A/Puerto Rico/8/1934 (H1N1; abbreviated as PR8) was kindly provided by Prof. Shihui Sun (Beijing Institute of Microbiology and Epidemiology) and propagated in 9- to 11-day-old SPF chicken embryos. The allantoic fluid was collected and titrated to determine the 50% tissue culture infection dose (TCID_50_) in A549 cells and the median lethal dose (LD_50_) in mice following the Reed-Muench method (Reed and Muench, 1938).

Specific pathogen free (SPF) grade female BALB/c mice aged 6–8 weeks (body weight: 18–20 g) were purchased from the Experimental Animal Center of the Military Medical Research Institute.

### Construction of a Cellular Model for the Overexpression/Inhibition of IGF1

Amplification of human IGF1 open reading frame (ORF; Guangzhou GeneCopoeia Biotechnology Co., Ltd.) using primers containing Xba I and Xho I restriction sites (Forward: 5′-TGCTCTAGAATGGGAAAAATCAGCAGTCT-3′; Reverse: 5′-CCGCTCGAGCTACATCCTGTAGTTCTTGT-3′) ligated into a pcDNA3.1 expression vector, constructing pcDNA3.1-IGF1. The pcDNA3.1-IGF1 vector was transfected into A549 cells with LiPO2000. The cell line overexpressing IGF1 was screened with G418 (500 μg/ml). The human IGF1 shRNA lentiviral particles (sc-37193-V) were purchased from Santa Cruz Company.

### mRNA Levels Detected by Real-Time Quantitative PCR

The total cellular RNA was extracted using TRIZOL (Invitrogen, Cat: 15596-026). The cDNA was synthesized by reverse transcription using a TIANscript RT Kit (TIANGEN, Cat: KR104), followed by quantitative PCR (qPCR) using SYBR Premix Ex Taq II (TAKARA, Cat: RR820A). The primer sequences that were used are presented in Table 1. When detecting the viral proliferation in the lungs of mice, a real-time fluorescent quantitative PCR probe method was used, and the probe sequence was FAM-TGCAGTCCTCGCTCACTGGGCACG-BHQ1. The primer sequence of matrix protein 1 (M1) was Forward: 5′-GACCRATCCTGTCACCTCTGAC-3′; Reverse: 5′-GGGCATTYTGGACAAAKCGTCTACG-3′. GAPDH was selected as the internal reference, and the results were analyzed using the 2^−△△Ct^ method. The reaction conditions were set as follows: step 1: 95°C for 30 s; step 2: 95°C for 5 s, 60°C for 30 s, 40 cycles; and step 3: dissolution curve analysis.

**Table 1:** Quantitative PCR primer sequences for inflammatory cytokines.

Gene	Primer 5′-3′
GAPDH-F	AACGGGAAGCTCACTGGCATG
GAPDH-R IGF1-F IGF1-R	TCCACCACTGTTGCTGTAG TAAGGAGGCTGGAGATGTAT CTCTACTTGCGTTCTTCA
IL-6-F	AGCCCTGAGAAAGGAGACATG
IL-6-R	GCAAGTCTCCTCATTGAATCCAG
IL-8-F	TGTGGAGAAGTTTTTGAAGAGGG
IL-8-R	CCCTACAACAGACCCACACAATAC
IL-10-F	ACCTGCCTAACATGCTTCGAG
IL-10-R	CTGGGTCTTGGTTCTCAGCTT
TNF-α-F	TGGAGAAGGGTGACCGACTCAG
TNF-α-R	GTTTGGGAAGGTTGGATGTTCG
IL-1β-F	CCCAGAGAGTCCTGTGCTGAATG
IL-1β-R	GAGAGCTGACTGTCCTGGCTGAT
CCL2-F	GCTCGCTCAGCCAGATGCAATC
CCL2-R	GCTTCTTTGGGACACTTGCTGC

### Western Blot Detection of the Level of Protein Expression

For the cultured cells, the total cellular protein was extracted using the Whole Cell Lysate (Beijing ComWin, Cat: CW0074) containing a protease inhibitor cocktail (Roche, Germany). The protein was quantified using a bicinchoninic acid (BCA) Protein Assay Kit (Beijing ComWin, Cat: CW0014), and a 30 μg protein sample was obtained for polyacrylamide gel electrophoresis (PAGE). The lung tissues from six mice of each group were mixed and ground in liquid nitrogen, and then the total cellular protein was extracted using the Whole Cell Lysate (Beijing ComWin, Cat: CW0074) containing a protease inhibitor cocktail (Roche, Germany). Protein was quantified using a BCA Protein Assay Kit (Beijing ComWin, Cat: CW0014). A 50 μg protein sample was obtained for PAGE and subsequently transferred to a polyvinylidene fluoride (PVDF) membrane. The primary antibodies of rabbit anti-glyceraldehyde phosphate dehydrogenase (GAPDH) and goat anti-IGF1 (Abcam) were diluted to 1:2,000. The secondary antibodies of horseradish peroxidase (HRP)-goat anti-rabbit immunoglobulin G (IgG) and HRP-rabbit anti-goat IgG (ZSGB-BIO) were diluted to 1:5,000. The signals were detected using a Western HPR Substrate Peroxide solution (Millipore). The quantification of Western blot analysis was performed by using Image J software, and the protein expression levels were normalized to GAPDH levels.

### Level of Cytokine Expression Detection

The level of cytokine expression was detected using mouse IGF1 ELISA Kit, IL-6 ELISA Kit, TNF-α ELISA Kit, IFN-γ ELISA Kit, and IL-1β ELISA Kit (all purchased from R&D systems).

### Viral Infection of A549

For viral infection, A549 cells were washed with phosphate buffered saline (PBS) and subsequently infected with PR8 at the multiplicity of infection (MOI) of 0.5 or 1.0 in infection medium, which was DMEM supplemented 0.5 mg/ml N-p-tosyl-phenylalanine chloromethyl ketone (TPCK)-treated trypsin (Sigma, USA) and 0.3% bovine serum albumin (BSA; Sangon, China). After 1 h, cells were incubated with fresh infection medium at 37°C for the indicated times.

### Infection, Monitoring, and Sampling of Mice

BALB/c mice were intraperitoneally injected with sodium pentobarbital (50 mg/kg), and the mice were induced into a deep anesthetic state and administered a nasal inhalation of phosphate buffered saline (PBS) or PR8 (diluted 10-fold, 30 μl/mouse, 50LD_50_). In the PR8 + IGF1 group, IGF1 (20 μg/kg, Peprotech) was intraperitoneally injected 6 h before infection, and after infection, IGF1 was intraperitoneally injected every 24 h. The PR8 + PPP group was intraperitoneally injected with the IGF1 receptor inhibitor, and picropodophyllin (PPP, 20 mg/kg, Selleck) 6 h before infection and after infection PPP was intraperitoneally injected every 12 h. After the continuous administration for 14 days, changes in the clinical symptoms of mice were observed daily, and changes in the body weight and survival were recorded. On days 3, 5, 7, and 9 after PR8 infection, six mice of each group were sacrificed every time. The blood was taken by excising the eyeballs, and the lung tissues were collected. The blood was kept at room temperature for 1 h and centrifuged at 11,000 *g* for 15 min. The serum was collected and aliquoted and stored at −80°C for later use.

All animal experimental procedures were approved by the Animal Care and Use Committee of the Academy of Military Medical Sciences (AMMS; ID: SYXK2012-05) and were carried out in strict accordance with the guidelines. All experiments involving the live virus were performed in an approved biosafety level 2 facility.

### Lung Injury Conditions and Lung Index

After removing the whole lung tissue of the mice, damage to the lung tissue was observed. The degree of lung injury visible to the naked eye was dark red due to edema. The area ratio of lung injury to the total lung tissue was estimated. Each sample was estimated by at least three different individuals, and the average was obtained. Finally, the lung injury area of six mice in each group was counted. The wet weight of the lung tissue was weighed. Lung index = lung wet weight/body mass.

### Data Analysis

All experiments were repeated at least three times. Data were expressed as means ± standard deviation (SD). The graphical representation of the data was performed using GraphPad Prism 5 software. The grayscale analysis of the Western blot images was performed using Image J software by comparing the integrated density of the IGF1 band with the control GAPDH. The statistical analyses were performed using SPSS 20.0 statistical software with two-tailed Student’s *t* test for two comparisons, *p < 0.05; **p < 0.01; and ***p < 0.001. A *p* below 0.05 was considered statistically significant.

## Results

### IGF1 Expression Is Upregulated in A549 Cells Infected With Influenza A Virus PR8

**Figure 1:**
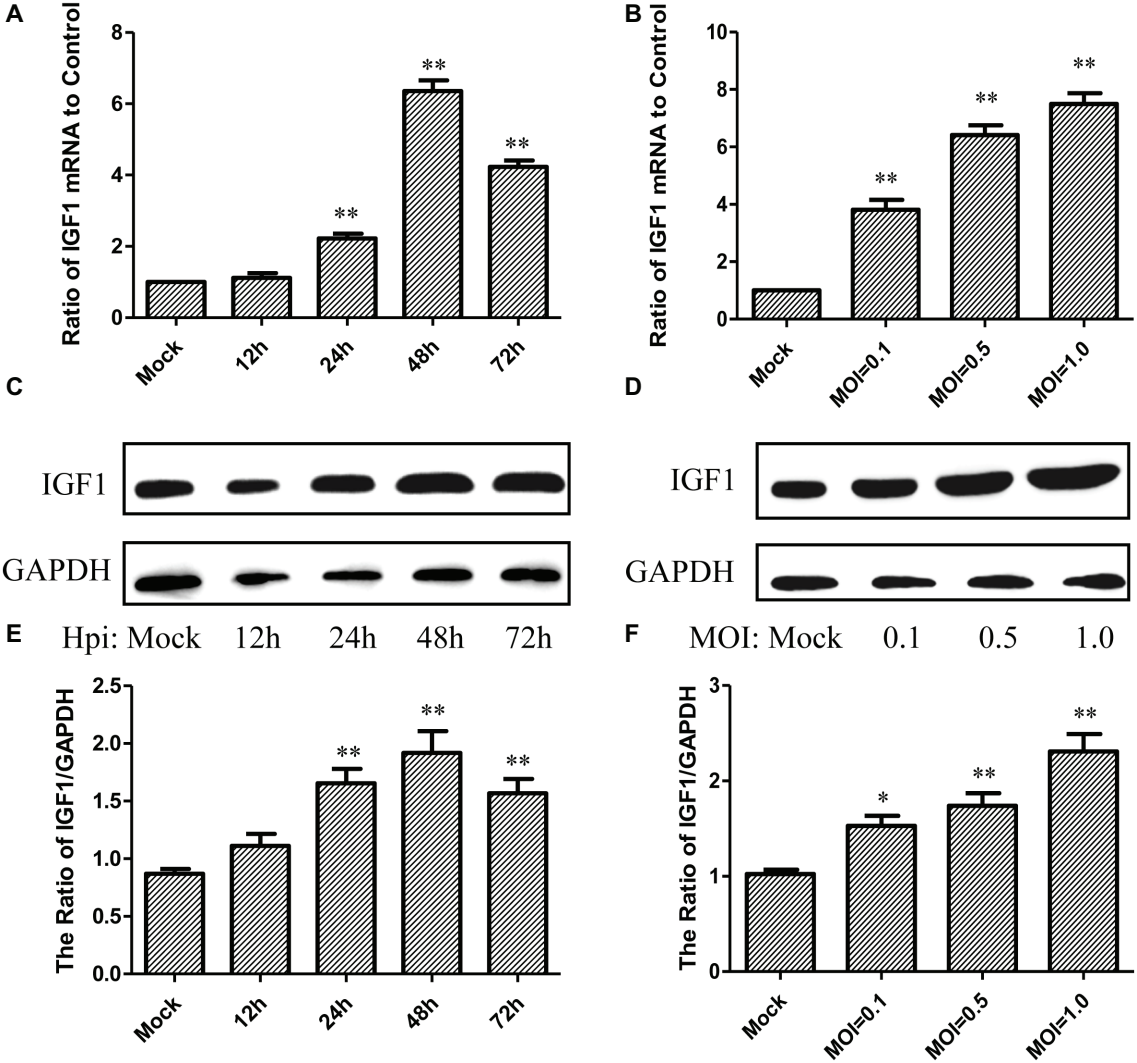
IGF1 expression was upregulated in A549 cells infected with influenza A virus (IAV) PR8. **(A)** Changes in the level of IGF1 mRNA over time in PR8-infected A549 cells (MOI = 0.5); **(B)** changes in the level of IGF1 mRNA over MOI in PR8-infected A549 cells at 48 h post-infection; **(C)** changes in the level of IGF1 protein expression over time in PR8-infected A549 cells (MOI = 0.5); GAPDH was used as loading control, and figures were representative of three independent experiments; **(D)** changes in the level of IGF1 protein expression over MOI in PR8-infected A549 cells at 48 h post-infection; GAPDH was used as loading control, and figures were representative of three independent experiments; **(E)** grayscale analysis of Figure 1C, and plotted as the ratio of the integrated density of IGF1/GAPDH; **(F)** grayscale analysis of Figure 1D, and plotted as the ratio of the integrated density of IGF1/GAPDH. Data were the means ± SD from three independent experiments. **p < 0.01 vs. Mock by *t* test.

To detect the regulation of IGF1 by IAV, A549 cells were infected with PR8 (10^4.58^ TCID_50_/ml) at different multiplicity of infection (MOI) of 0.1, 0.5, or 1.0, and the level of IGF1 mRNA and protein expression was detected at 12, 24, 48, and 72 h post-infection. As shown in Figure 1A, compared with the Mock-infected controls, the level of IGF1 mRNA expression in A549 cells increased gradually at 12, 24, and 48 h after PR8 infection at a MOI of 0.5. The level of IGF1 mRNA expression peaked at 48 h post-infection, which was 6.35 ± 0.30 times higher than that of the Mock controls. The level of IGF1 mRNA expression decreased slightly at 72 h post-infection but still reached 4.23 ± 0.18 times that of the Mock group. As shown in Figure 1B, in A549 cells infected with different MOIs of PR8, the level of IGF1 mRNA expression increased following the increasing of MOI at 48 h post-infection. At a MOI of 1.0, the level of IGF1 mRNA increased to 7.49 ± 0.38 times that of the Mock group.

The level of IGF1 protein expression was also upregulated at 12, 24, 48, and 72 h post-infection of PR8 (Figure 1C) and following the increasing of MOI at 48 h post-infection in A549 cells (Figure 1D). To compare the trend in IGF1 variability more intuitively, the grayscale analysis of Figures 1C,D was performed and plotted according to the ratio of the integrated density of IGF1/GAPDH. Figures 1E,F show that the level of IGF1 protein expression in the A549 cells following PR8 infection was consistent with the level of IGF1 mRNA; however, such upregulation times were lower than the level of mRNA. Compared with the Mock group, the level of IGF1 protein expression peaked 1.82 times at 48 h after PR8 infection at a MOI of 0.5 (Figure 1E). The level of IGF1 protein expression was also increased following the increasing of MOI. At a MOI of 1.0, the level of IGF1 protein expression reached 2.26 times that of the Mock group at 48 h post-infection (Figure 1F). The above results indicate that IGF1expression is upregulated in PR8-infected A549 cells.

### IGF1 Regulates the Expression of Inflammatory Cytokines in Response to PR8 Infection

**Figure 2:**
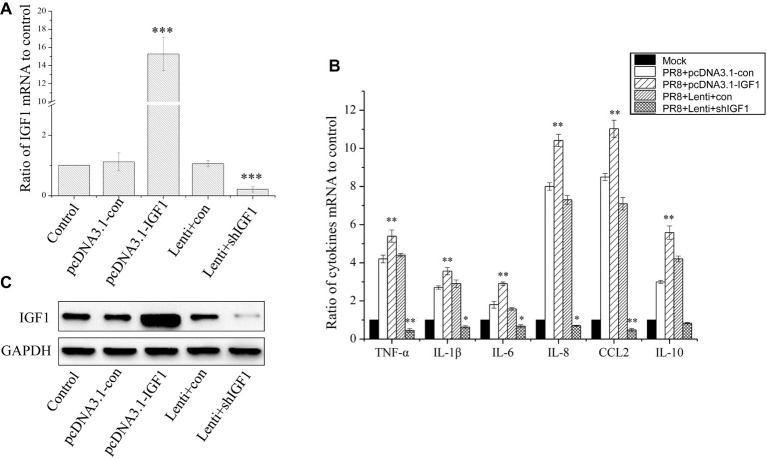
IGF1 regulates the expression of inflammatory cytokines in response to PR8 infection. **(A)** Q-PCR detection of IGF1 mRNA overexpression and inhibition in stably transfected A549 cells; **(B)** Western blot detection of IGF1 overexpression and inhibition in stably transfected A549 cells; GAPDH was used as loading control, and figures were representative of three independent experiments; **(C)** regulation of the intracellular inflammatory cytokines expression following PR8 infection (MOI = 0.5) after overexpression or inhibition of IGF1. Data were the means ± SD from three independent experiments. *p < 0.05; **p < 0.01; ***p < 0.001 vs. Mock by *t* test.

To investigate the role of IGF1 in the inflammatory response to influenza virus infection, we established two stable A549 cell lines, in which IGF1 is overexpressed or inhibited. Using pcDNA3.1-IGF1 to overexpress IGF1, Figure 2A showed that the level of IGF1 mRNA was increased 15.28 ± 1.84 times that of the control group. The expression of IGF1 in the cells was inhibited by lentiviral packaged shRNA (Lenti + shIGF1), after which the level of IGF1 mRNA was reduced to 0.21 ± 0.087 times that of the control group (Figure 2A). As shown in Figure 2B, the level of IGF1 protein was also increased in stable A549 cell line with pcDNA3.1-IGF1 and inhibited in stable A549 cell line with Lenti + shIGF1. The stably transfected A549 cell lines were infected with PR8 at a MOI of 0.5. After 24 h, real-time quantitative PCR (qPCR) was used to detect the changes in cytokine expressions [IL-6, IL-8, IL-10, monocyte chemotactic protein-1 (MCP-1, or CCL2), TNF-α, and IL-1β], which were related to the inflammatory response. The results in Figure 2C showed that compared with the Mock controls, the cytokine levels were significantly increased following PR8 infection in control cells (PR8 + pcDNA3.1-con group and PR8 + Lenti + con group). IGF1 overexpression further increased the level of cytokine mRNA expression, whereas the levels of cytokine expression in the PR8-infected cells with IGF1 knocked down (PR8 + Lenti + shIGF1) were significantly decreased far lower than the PR8-infected control cell (PR8 + Lenti + con group), as well as the uninfected Mock group. Especially the lowest CCL2 and TNF-α mRNA of PR8 + Lenti + shIGF1 group were downregulated to less than half that of the Mock group. These results indicate that the overexpression of IGF1 aggravated the inflammatory response induced by influenza virus infection, whereas the inhibition of IGF1 expression can alleviate this inflammatory response.

### IGF1 Expression Is Upregulated in Mice Infected With IAV PR8

**Figure 3:**
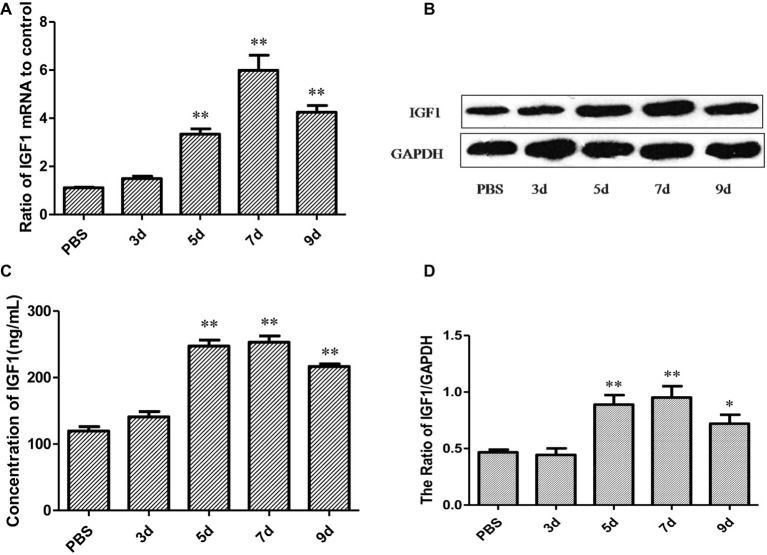
IGF1 expression is upregulated in BALB/c mice infected with IAV PR8. **(A)** Changes in the level of IGF1 mRNA over time in the lung tissue of BALB/c mice infected with PR8 (50LD_50_); **(B)** changes in the level of IGF1 protein over time in the lung tissue of BALB/c mice infected with PR8 (50LD_50_); **(C)** grayscale analysis of Figure 3B, and plotted as the ratio of the integrated density of IGF1/GAPDH; and **(D)** changes in the level of IGF1 mRNA expression over time in the serum of BALB/c mice infected with PR8 (50LD_50_). The lung and serum sample were mixed from six mice in the group. Data were the means ± SD from three independent experiments. **p < 0.01 vs. PBS group by *t* test.

To detect whether influenza virus can also regulate IGF1expression *in vivo*, IAV PR8 (diluted 10-fold, 30 μl/mouse, 50LD_50_) was intranasally inoculated into BALB/c mice. Mice were sacrificed on days 3, 5, 7, and 9 post-infection to obtain mice lung tissue and serum, and the samples of six mice in every group were mixed. The level of IGF1 mRNA expression in the lung tissue of mice was detected by qPCR. As shown in Figure 3A, the level of IGF1 mRNA expression in the lung tissue gradually increased at days 3, 5, and 7 after PR8 infection. On day 7 post-infection, IGF1 mRNA increased to 5.81 ± 0.623 times that of the control group and decreased slightly on day 9 post-infection. A Western blot was used to detect changes in IGF1 protein expression in the lung tissue following PR8 infection (Figure 3B). The grayscale analysis by comparing the integrated density of the IGF1 band with the control GAPDH showed that the trend in the level of IGF1 protein expression was consistent with that of the mRNA data (Figure 3C). The content of IGF1 in the serum of mice was detected by enzyme linked immunosorbent assay (ELISA). As shown in Figure 3D, compared with the PBS control group, the content of IGF1 protein in serum increased significantly on day 5 post-infection of PR8 and decreased on day 9. These results were also consistent with the qPCR and Western blot findings.

### IGF1 Regulates Acute Inflammatory Lung Injury in PR8-Infected Mice

To detect whether IGF1 regulates acute inflammatory lung injury in PR8 infection, BALB/c mice were tested as follow. BALB/c mice were randomly divided into four groups: (1) PBS; (2) PR8 + IGF1; (3) PR8+ PPP; and (4) PR8 + PBS. The PBS group was intranasally inoculated with 30 μl PBS, whereas the other three groups were intranasally inoculated with 30 μl influenza virus PR8 (50LD_50_). From 6 h before the inoculation to day 14 post-infection, the mice in the PR8 + IGF1 group were intraperitoneally injected with IGF1 protein (20 μg/kg/24 h); PR8 + PPP mice were injected intraperitoneally with an IGF1 receptor inhibitor, picropodophyllin (PPP, 20 mg/kg/12 h); and the PR8 + PBS group was injected with PBS (30 μl/24 h). The clinical symptoms of the mice were observed daily for 14 days, and changes in body weight and survival were recorded. Six mice of each group were sacrificed at day 5 post infection of PR8, and the serum and lung tissues of each group were collected and mixed. The extent of lung injury and inflammatory cell infiltration of the mice in each group was observed by histologically. Changes in the level of serum cytokines were detected by ELISA. A western blot was used to detect the changes in the expression of major proteins in the IGF1/IGF1R-related signaling pathways.

**Figure 4:**
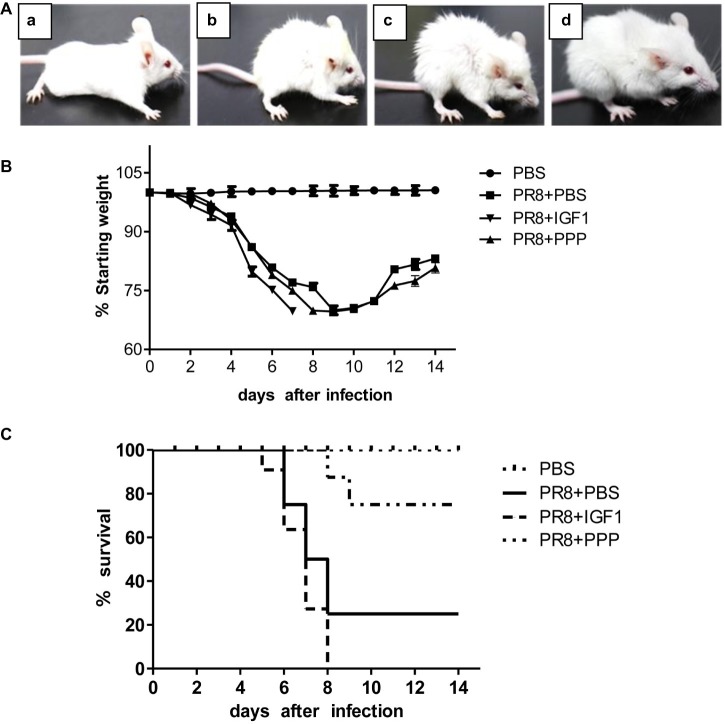
Changes in the clinical manifestations, body weight, and survival rate of BALB/c mice following PR8 infection. **(A)** Clinical symptoms of BALB/c mice after PR8 infection (50LD_50_) [**(a)** PBS; **(b)** PR8 + PBS; **(c)** PR8 + IGF1; **(d)** PR8 + PPP]; **(B)** changes in body weight in BALB/c mice after PR8 infection (50LD_50_); and **(C)** survival rate of BALB/c mice after PR8 infection (50LD_50_; *n* = 15).

There was no change in the general appearance of the PBS control group mice. In the PR8 + PBS group, flu-like symptoms began to appear on day 3 after PR8 inoculation, such as reduced activity, ruffled fur, and slight weight loss. On day 5 post-infection, these conditions worsened with 20% weight loss, canthus secretion, hunched back. The symptoms of the PR8 + IGF1 group were more severe than those of the PR8 + PBS group, whereas the PR8 + PPP group displayed milder clinical symptoms than that of the PR8 + PBS group (Figure 4A). Figure 4B showed that the body weight of the mice decreased significantly on day 3 post-infection of PR8, and the body weight of the mice on day 9 decreased to the lowest. Although the changes in body weight were similar between the PR8 + PPP group and PR8 + PBS group, the survival rate of the PR8 + PPP group was dramatically increased up to 75% compared with the survival rate of the PR8 + PBS group was only 25%, with the first death not occurring until the day 8 post-infection (Figure 4C). The first death in the PR8 + IGF1 group was accelerated to day 5 post-infection, and all mice have succumbed to the infection by day 8. These findings indicated that the intraperitoneal injection of IGF1 promoted the death of PR8-infected mice, whereas the inhibition of IGF1 increased the survival rate of PR8-infected mice.

**Figure 5:**
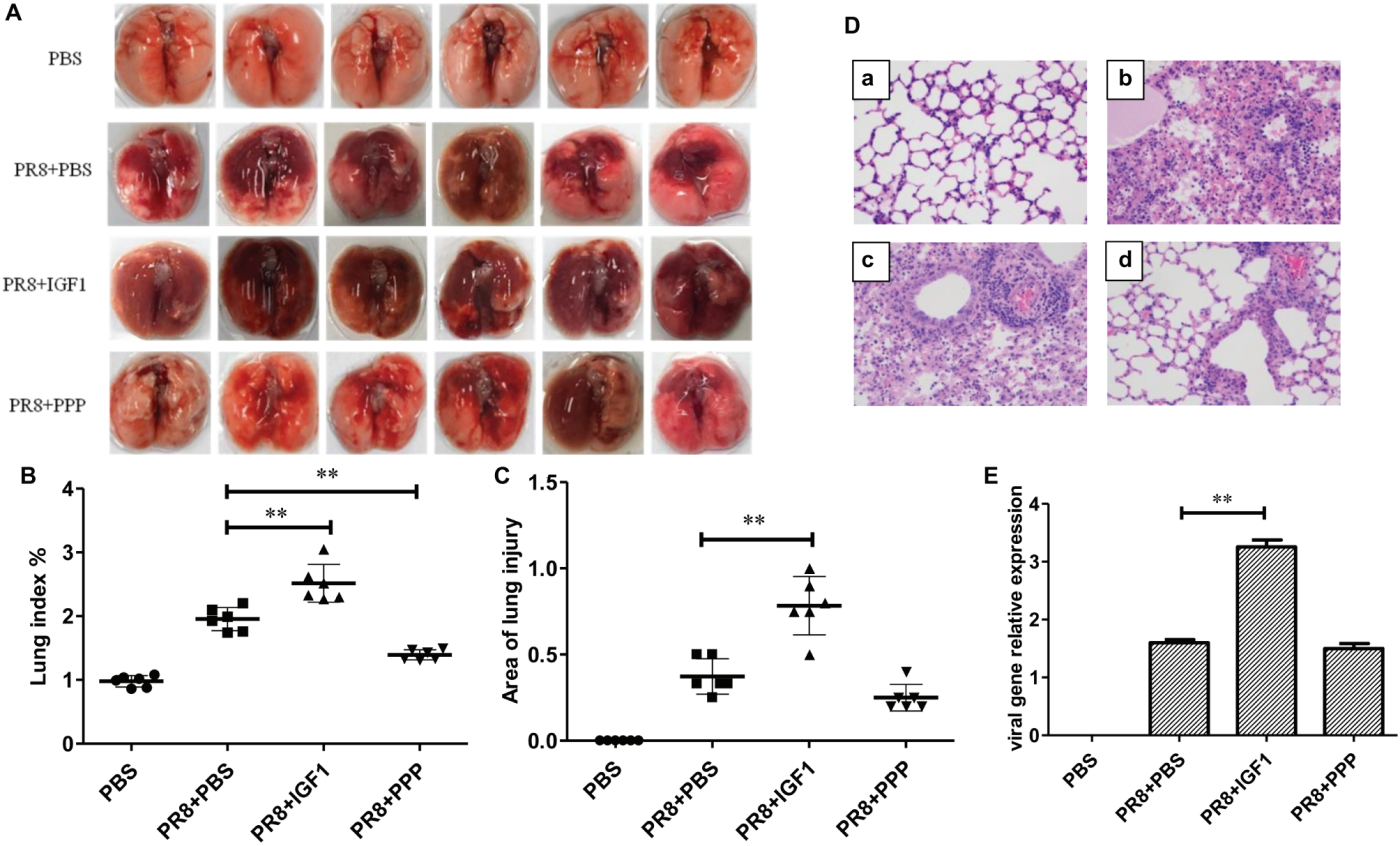
Lung injury following PR8 infection in BALB/c mice. Groups of BALB/c mice (*n* = 6, females, 6–8 weeks) were intranasally infected with PR8 (50LD_50_), treated with IGF1 or PPP, and at day 5 post-infection, the mice lungs were examined for changes in **(A)** morphology, **(B)** lung injury, **(C)** injury areas, **(D)** histopathology, and **(E)** viral load. Data were the means ± SD from three independent experiments. **p < 0.01 vs. PR8 + PBS group by *t* test.

Figure 5A showed that in IAV PR8-infected mice, the lungs exhibited differential degrees of damage, and the color of the damaged parts changed from pink to dark red, with the presence of edema. The extent of lung injury in the PR8 + IGF1 group was significantly more severe than that of the PR8 + PBS group, as the lung color was darker, and the lesion area was larger. The degree of lung injury in the PR8 + PPP group was significantly less severe than that of the PR8 + PBS group. Figures 5B,C showed that the lung index of uninfected mice was approximately 1%. Compared with the PR8+ PBS group, the lung index of the PR8 + IGF1 group increased significantly from 1.953 ± 0.074 to 2.515 ± 0.121%, and the area of lung injury also increased from 0.38 ± 0.042 to 0.78 ± 0.069, approximately twice that of the control group. In the PR8 + PPP group, the lung index decreased to 1.393 ± 0.032%, and the lung injury area also decreased to 0.25 ± 0.062, which were both lower than that of the PR8 + PBS group. As shown in Figure 5D, the lung structure of the mice in the PBS group was clear, with intact pulmonary alveoli, clear alveolar septum, and without hemorrhaging and inflammatory cell infiltration. The pulmonary alveoli of the PR8 + PBS, PR8 + IGF1, and PR8 + PPP groups exhibited varying degrees of damage. The level of inflammatory cell infiltration in the lung tissue of the PR8 + IGF1 group was greater than that of the PR8 + PBS group, and there was lower inflammatory infiltration in the PR8 + PPP group than the PR8 + PBS group.

Whether IGF1 regulates the viral replication of IAV, qPCR was used to detect changes of the viral matrix protein 1 (M1) expression in the lungs of PR8-infected mice, which could indirectly reflect the viral load. The viral load in the lungs of the PR8 + IGF1 group was significantly increased to nearly two-fold of the PR8 + PBS group (Figure 5E). However, the viral load of the PR8 + PPP group and the PR8 + PBS group was similar, indicating that IGF1 responded to influenza infection *via* the host immune response rather than targeting viral replication.

**Figure 6:**
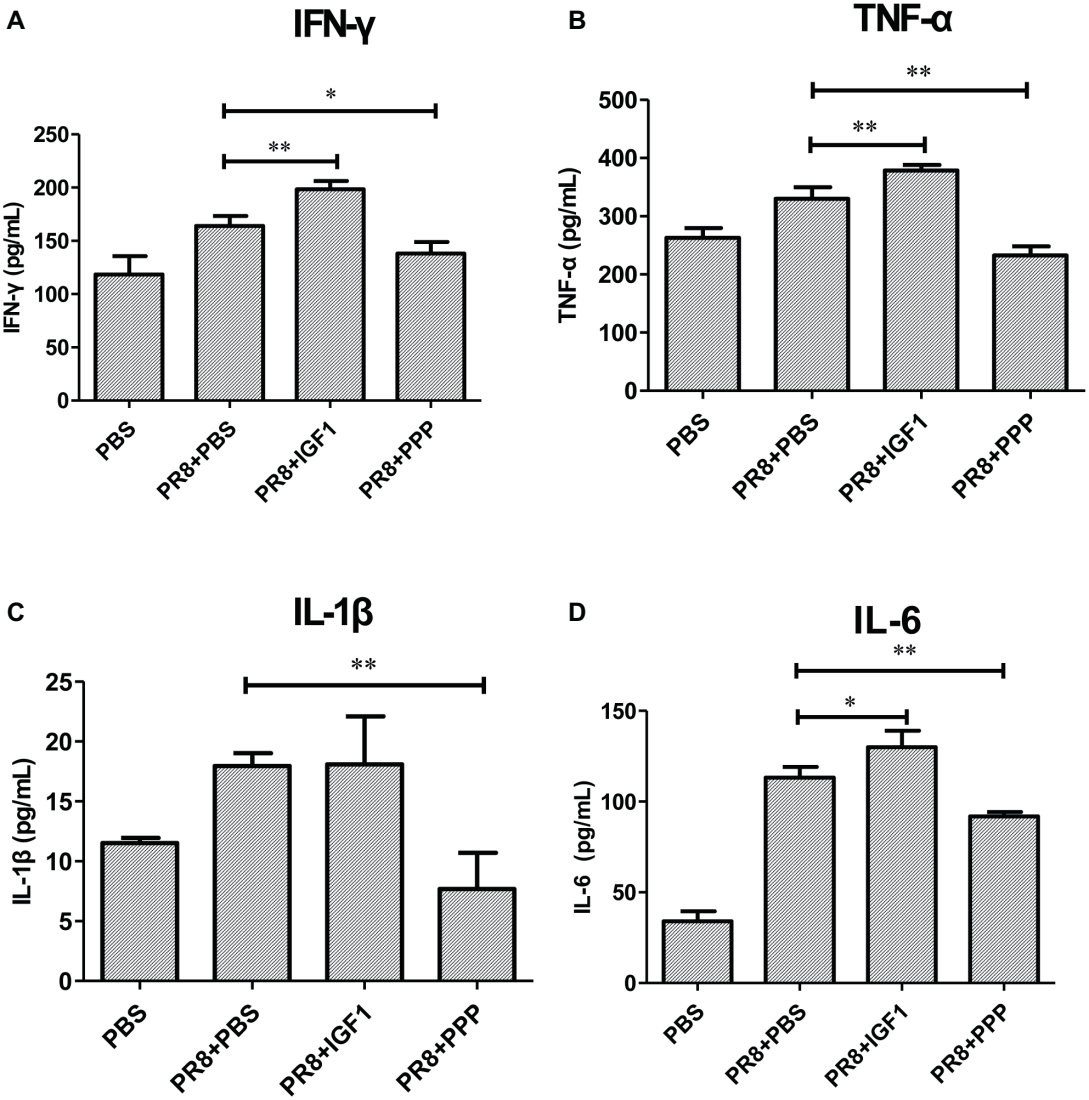
Cytokine expression in BALB/c mice after PR8 infection. Groups of BALB/c mice (*n* = 6, females, 6–8 weeks) were intranasally infected with PR8 (50LD_50_), treated with IGF1 or PPP, and at day 5 post-infection, the mice serum were examined for changes in **(A)** IFN-γ mRNA, **(B)** TNF-α mRNA, **(C)** IL-1β mRNA, and **(D)** IL-6 mRNA. Data were the means ± SD from three independent experiments. *p < 0.05; **p < 0.01 vs. PR8 + PBS group by *t* test.

The cytokine content in the serum of PR8-infected mice was detected. Figures 6A–D showed that there was a significant increase in the serum inflammatory following PR8 infection. Moreover, the inflammatory cytokine levels in the intraperitoneal injected IGF1 protein group were further increased, in which IL-1β was highly variable, and there was no statistical difference with the PR8 + PBS group. The levels of inflammatory factors IFN-γ, TNF-α, IL-6, and IL-1β in the serum of the PR8 + PPP group were significantly decreased, and IL-1β was lower than that of the uninfected mice. The results further confirm that IGF1 induced an inflammatory response following influenza virus infection, which can be alleviated by inhibiting IGF1.

### IGF1 Regulates Acute Inflammatory Lung Injury in PR8-Infected Mice *via* the PI3K/AKT and MAPK Pathways

**Figure 7:**
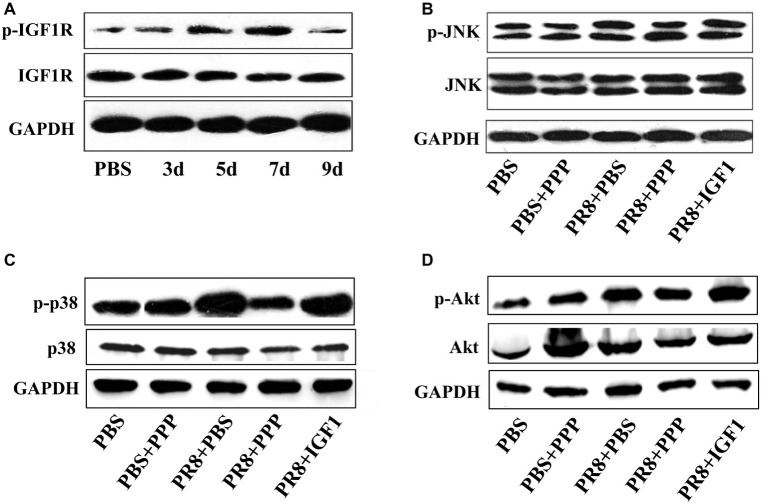
Changes in the level of protein expression in the IGF1 signaling pathway. **(A)** Changes in the level of IGF1R and p-IGF1R protein over time in the lung tissue of BALB/c mice infected with PR8 (50LD_50_); groups of BALB/c mice (*n* = 6, females, 6–8 weeks) were intranasally infected with PR8 (50LD_50_) treated with IGF1 or PPP, and at day 5 post-infection, the mice lungs were examined for changes in **(B)** JNK and p-JNK protein, **(C)** p38 and p-p38 protein, and **(D)** Akt and p-Akt protein. The lung sample was mixed from six mice in the group. Figures were representative of three independent experiments.

To explore the mechanism by which IGF1 regulates acute inflammatory lung injury induced by IAV infection, a Western blot was used to detect the expression of molecules in key signaling pathways in the lung tissue of PR8-infected mice (50LD_50_ PR8). Each sample was from six mice in a group and was tested for three times. Figures were representative of three independent experiments. Figure 7A showed that the phosphorylation level of IGF1R increased gradually following PR8 infection, peaked at day 7 post-infection, and then decreased slightly on day 9, which was consistent with the changes in the trend of IGF1 protein expression (Figure 3B). Figures 7B–D showed that the PI3K/AKT and MAPK signaling pathways were activated following PR8 infection; p-AKT expression was upregulated in the PI3K/AKT signaling pathway; and p-p38 and p-JNK expression were upregulated in the MAPK signaling pathway. The levels of p-AKT and p-p38 expression were further increased in the PR8 + IGF1 group. The expression level in the PR8 + PPP group was between that of the PBS and PR8 + PBS groups. There was no significant difference in the level of p-JNK expression between the groups infected with influenza virus. The results indicated that the administration of IGF1 protein following influenza virus infection promoted the activation of the PI3K/AKT and MAPK signaling pathway. Intervention with the IGF1R inhibitor PPP inhibited influenza virus mediated PI3K/AKT and MAPK signaling pathways. These results suggest that IGF1 affected the expression of key proteins associated with MAPK and the PI3K/AKT signaling pathway in response to IAV-mediated inflammation.

## Discussion

In this study, we investigated that IGF1 played a significant role in mediating inflammation and pathology during IAV infection. IGF1 expression was upregulated in A549 cells and BALB/c mice infected with PR8, which modulated inflammatory cytokine expression. IGF1 overexpression aggravated influenza-mediated inflammatory responses, whereas the inhibition of IGF1 expression reduced such inflammatory responses. The phosphorylation of IGF1R triggered the PI3K/AKT and MAPK signaling pathways to induce inflammation. Thus, inhibiting IGF1 or IGF1R expression to block downstream signaling may be a novel treatment strategy for influenza infection.

Following an influenza virus infection, a series of immunological responses are initiated. First, the host’s innate immune system, consisting of interferon, cytokines, macrophages, neutrophils, NK cells, and Dendritic cells (DCs), all rapidly respond to the viral infection and activate adaptive immunity (Gu et al., 2019). Although the innate immune response is important, the host typically eliminates the viral infection with the aid of the adaptive response (Bonilla and Oettgen, 2010). In humoral immunity, neutralizing antibodies can prevent the adsorption of the virus, *via* opsonization, and primarily interact with extracellular free viruses (Murasko et al., 2002). Activated Th1 cells release various cytokines (i.e., IFN-γ and TNF), activate macrophages and NK cells to induce further inflammatory reactions, and promote the proliferation and differentiation of cytotoxic T lymphocytes (CTLs), which play an important role in antiviral infection (Julkunen et al., 2001; Farsakoglu et al., 2019). However, studies have shown that while an inflammatory response helps to clear pathogens, excessive inflammatory reactions do not only protect the host organism but also cause the additional damage. Many studies have shown that the cause of death from an influenza virus infection is the pulmonary respiratory syndrome induced by inflammation caused by an excessive reaction of the body’s immune system (Blach-Olszewska and Leszek, 2007).

In the present study, changes in the level of IGF1 mRNA and protein expression in A549 cells were detected. The results showed that IGF1 mRNA and protein expression were significantly altered following IAV infection. To further confirm the important role of IGF1 in IAV-mediated inflammation and the associated signaling mechanism, IGF1 protein was administered as an intervention to PR8-infected mice. Compared with the untreated infected mice, the infected mice administered IGF1 displayed more severe clinical symptoms; in particular, the serum levels of IFN-γ, TNF-α, and IL-1β were significantly increased, and lung damage was more serious. IGF1 is known to function by binding to the receptor, IGF1R. In this study, the IGF1R inhibitor PPP was used to inhibit IGF1R function, thereby disrupting its downstream signaling capacity. By monitoring the clinical symptoms and inflammation indicators in mice, it was found that during the same period of viral infection, when PPP was used to inhibit IGF1, the clinical symptoms of mice were alleviated; the lung injury area of the mice was reduced; the degree of lung injury was reduced; the lung index was significantly decreased; the serum levels of inflammatory cytokines IFN-γ, TNF-α, IL-1β, and IL-6 were significantly decreased; and the survival rate increased from 25 to 75% compared with the untreated group. The viral proliferation in the lungs of mice treated with IGF1 was significantly increased; however, the viral proliferation of the mice treated with PPP was similar to that of the PR8 + PBS group. Thus, the inhibition of IGF1 only affects the immune response but has no significant effect on IAV replication. Thus, the inhibition of IGF1, rather than antiviral pathways that affect viral replication, may provide a new therapeutic avenue for influenza that limits detrimental inflammatory responses to infection. This is consistent with the previously reported effective treatments for influenza-mediated inflammation, which prolong the survival of older influenza-infected individuals, compared to those treated with antivirals (Pillai et al., 2016).

The inflammation-associated PI3K/AKT and MAPK signaling pathways are known to be activated in response to influenza virus infection (Dong et al., 2014; Hirata et al., 2014). Although IGF1 primarily regulates cell growth and apoptosis through the PI3K/AKT and MAPK signaling pathways, whether IGF1 plays a role in these pathways in the context of influenza virus-mediated inflammation remains unknown. In this study, a Western blot was used to detect the expression of key proteins in the PI3K/AKT and MAPK signaling pathways. The results showed that p-IGF1R was upregulated following PR8 infection; p-AKT expression was upregulated in the PI3K/AKT signaling pathway; and p-p38 and p-JNK expression were upregulated in the MAPK signaling pathway compared with the PBS group. The expression of p-AKT and p-p38 in the PR8 + IGF1 group was higher than that of the PR8 + PBS group, and the expression in the PR8 + PPP group was between that of the PBS and PR8 + PBS group. There was no significant difference in the level of p-JNK expression between the groups infected with influenza virus. This suggested that IGF1 affected the expression of key proteins associated with P38 in the MAPK and the PI3K/AKT signaling pathways in the context of influenza virus-mediated inflammation, which confirmed our hypothesis. Therefore, we conclude that IGF1 expression is upregulated following influenza virus infection, and IGF1 receptor phosphorylation is elevated, triggering two signaling pathways downstream of PI3K/AKT and MAPK to induce inflammation. Thus, the inhibition of IGF1 or IGF1R expression to block such downstream signaling pathways may represent a novel approach for the treatment of influenza virus infection.

## Data Availability Statement

The data used to support the findings of this study are available from the corresponding author upon request.

## Ethics Statement

The animal study was reviewed and approved by Academy of Military Medical Sciences (AMMS; ID: SYXK2012-05).

## Author Contributions

ChZ and GL contributed to conceptualization. ChZ, GL, and LZ contributed to methodology. LZ, CaZ, and YS contributed to investigation. RW and ChZ contributed to writing the original draft. RW and ChZ contributed to writing, reviewing, and editing. ChZ contributed to funding acquisition. LZ, DD, and MB contributed to resources. RW, GL, and ChZ helped in supervision.

### Conflict of Interest

The authors declare that the research was conducted in the absence of any commercial or financial relationships that could be construed as a potential conflict of interest.
